# Bibliometric Analysis of Global Trends in Research on Seasonal Variations in Gut Microbiota from 2012 to 2022

**DOI:** 10.3390/microorganisms11082125

**Published:** 2023-08-21

**Authors:** Jiancheng Zhai, Xiao Sun, Rui Lu, Xueqin Hu, Zhiqiang Huang

**Affiliations:** 1 Natural Reserve Planning and Research Institute, East China University of Technology, Nanchang 330013, China; 2 School of Earth Sciences, East China University of Technology, Nanchang 330013, China; 3 College of Animal Science and Technology, Jiangxi Agricultural University, Nanchang 330029, China

**Keywords:** gut microbiota, seasonal, bibliometric analysis, CiteSpace, visualization

## Abstract

Seasons are the important influencing factor for gut microbiota, which in turn affects the ecology and evolution of the host. The seasonal variation in gut microbiota has increasingly attracted the attention of researchers and professionals worldwide. However, studies of seasonal variations in gut microbiota have not been systematically analyzed by bibliometrics or visual analysis. This study is based on 271 publications from 2012 to 2022 in the Web of Science Core Collection database (WOSCC) to analyze hot spots and trends in this field. The collaborations between different countries, institutions, authors, journals, and keywords were bibliometrically analyzed using Excel, CiteSpace (Version 6.2. R4), and VOSviewer (version 1.6.19) software. The number of publications has been increasing rapidly and shows a general upward trend. China and the Chinese Academy of Sciences are the country and institution contributing the most, respectively. The research hotspots and trends mainly include the diversity of gut microbiota communities in different seasons, the relationship between diet and gut microbiota in seasonal changes, and the relationship between gut microbiota and evolutionary adaptation in seasonal changes. This is the first bibliometric and visualization analysis of seasonal variations in gut microbiota, which may advance this field and lay the foundation for future research.

## 1. Introduction

The gut microbiota has a close mutualistic symbiotic relationship with the host’s ecology and evolutionary outcomes [1]. The complex and variable micro-ecosystem of gut microorganisms is involved in metabolism, immune regulation, intestinal development promotion, pathogen defense, and other physiological activities [2,3], and even in many pathological conditions of several organ systems, including the genito-urinary system [4]. However, the gut microbiota is not static. It is influenced by biotic and abiotic factors, such as genetics, diet, gender, season, and the physiological state of the host [1,5,6]. For instance, changes in food composition exert strong selection pressures on the structure of gut microbiota [7,8]. Therefore, the gut microbiota of the same species can vary greatly at different times and in different environments, which can help the host to adapt to its surroundings by influencing host energy metabolism or other aspects.

Seasons are an important influencing factor for gut microbiota. The gut microbiota of animals responds quickly to seasonal changes, possibly to buffer the energy challenges generated by seasonal changes [9,10]. During the process of seasonal changes, forest squirrels shift from being mainly insect eaters to seed eaters, with a gradual decrease in the proportion of *Lactobacillus* in the gut microbiota, while the proportion of *Alistipes* and *Helicobacter* shows a significant increase. This discovery provides strong evidence for the view that changes in the composition of host gut microbiota are related to seasonal adaptation [11]. Meanwhile, similar seasonal variations in gut microbiota have been demonstrated in white-faced capuchin monkeys (*Cebus capucinus imitator*) [12], American bison (*Bison bison*) [13], Musk deer (*Moschus berezovskii*) [14], white-lipped deer (*Cervus albirostris*) [15], yaks (*Bos grunniens*) and Tibetan sheep (*Ovis aries*) [16]. The seasonal transformation of gut microbiota in composition and function can effectively help hosts adapt to changes in food supply and energy fluctuations, which also reflects the coevolutionary relationship between animals and gut microbiota in response to seasonal changes [17]. During the dry season, the simultaneous increase in the bacterial community and concentration of short chain fatty acids involved in fiber fermentation may cause the Mexican black howler monkey (*Alouatta pigra*) to maintain energy balance when energy is insufficient, without altering activity or range patterns [18]. Gut microbiota can enhance the intestinal absorption, energy homeostasis, and fat consumption abilities of mice during cold seasons, thereby improving the energy challenge of living in cold environments [19].

Seasonal variations in gut microbiota have increasingly attracted the attention of researchers and professionals worldwide. Therefore, creating and using web-based information solutions to navigate and process this kind of data is an urgent and necessary task for scientific research work. The knowledge graph comprises a new branch of scientific metrology. It can visually display the core structure, development history and frontiers of a discipline, and the overall knowledge structure to achieve the purpose of multidisciplinary integration through a series of processes, such as data mining, information analysis, scientific metrology, and graph drawing [20,21]. CiteSpace is a citation visualization literature analysis tool based on scientific metrology and data visualization theory, which can effectively display the development trend of a certain discipline or knowledge field in a certain period [22,23]. Thus, this study aims to use CiteSpace to (i) investigate the state of the art of research involving seasonal variations in gut microbiota in the last 10 years (from 2012 to 2022); (ii) understand the most influential publications in this field; (iii) identify the principal authors, institutions, and countries where the studies are developed; (iv) analyze the main keywords; and (v) identify future trends in this line of research.

## 2. Materials and Methods

### 2.1. Data Source

The original data for this study were obtained from the Web of Science Core Collection database (WOSCC), and were published from 1 January 2012 to 31 December 2022. The search formula used was “TI = (gut or intestin*) and TI = (microb* or flora or bacteria*) and AB = (spring or summer or autumn or winter or season*)”. The search date was March 16, 2023, and the language was limited to English. Article type was restricted to articles and reviews only. The literature retrieval was carried out independently by two researchers. Ultimately, excluding non-compliant articles, we identified 271 eligible records. Search records were downloaded and exported to CiteSpace (Version 6.2. R4, Drexel University, Philadelphia, PA, USA) software for subsequent analysis.

The journal impact factor (IF) and quartile were obtained from Journal Citation Reports 2023. The IF is determined by the number of citations and total articles in the last two years and reflects the journal’s influence [24].

### 2.2. Statistics and Analysis

The data were imported into Microsoft Excel (Version 2010, Redmond, Washington, DC, USA), GraphPad Prism (Version 6.0, GraphPad Prism Software Inc., San Diego, CA, USA), VOSviewer (Version 1.6.19, Leiden University, Leiden, The Netherlands), and CiteSpace (Version 6.2. R4). The study analyzed and visualized bibliometric information on the number of publications, publication geography, journals, authors, and keywords for each year from 2012 to 2022.

We analyzed the cooperation relationships of countries based on VOSviewer (Version 1.6.19) software and drew an international cooperation network diagram using Scimago Graphica (Version 1.0.35, SRG S.L. company, Granada, Spanish) software to present a comprehensive view of the geographic distribution of publications. Institutions, representing the distribution of research forces, were analyzed to identify prominent institutions and collaborative relationships. In the visualization network, nodes and links are included. Nodes represent countries, institutions, authors, references, etc., while links between nodes represent the relationship between them. The size and frequency of nodes are in direct proportion, and the thickness of the connecting lines implies the strength of the correlation. Nodes and links are represented in different colors in chronological order, while cooler colors (such as blue) represent older studies and warmer colors (such as red) represent newer studies. In addition, burst detection, which is an algorithm used to identify sudden changes in events and other types of information, was performed on keywords, and the red ring on the resultant image represents the start and end of the citation surge.

## 3. Results

### 3.1. Temporal Trends of Publications and Citations

**Figure 1:**
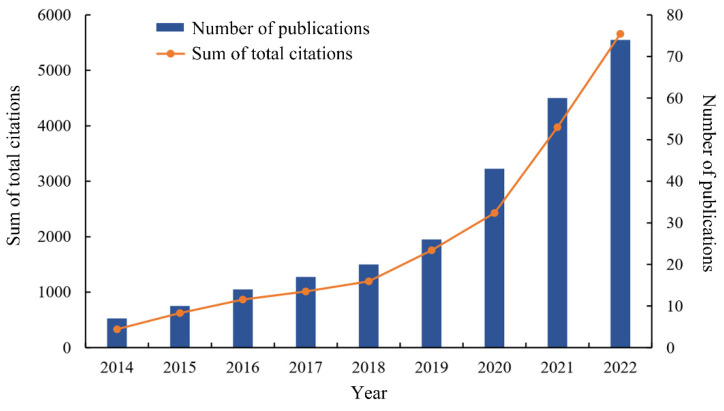
The trend in terms of publications and citations. Blue bars represent the number of publications related to the research on seasonal variations in gut microbiota, and the orange line represents the trend of total citations in this field.

We searched for seasonal variations in gut microbiota from 2012 to 2022 and found that there have been 271 articles in this field since 2014. The number of annual publications showed a general upward trend, and the number of publications increased by more than 10-fold, from 7 in 2014 to 74 in 2022 (Figure 1). The number of publications has grown the fastest in the past three years (2020–2022), accounting for the highest proportion (65.31%), while the initial three years (2014–2016) had the lowest proportion (11.44%), with the middle three years (2017–2019) accounting for 23.25%. The analysis found that the number of total citations was 17,484. With the rapid increase in the number of publications, the number of citations in publications continued to increase, indicating that the field of seasonal variations in gut microbiota had received increasing attention in recent years.

### 3.2. Countries and Institutions Analysis

**Figure 2:**
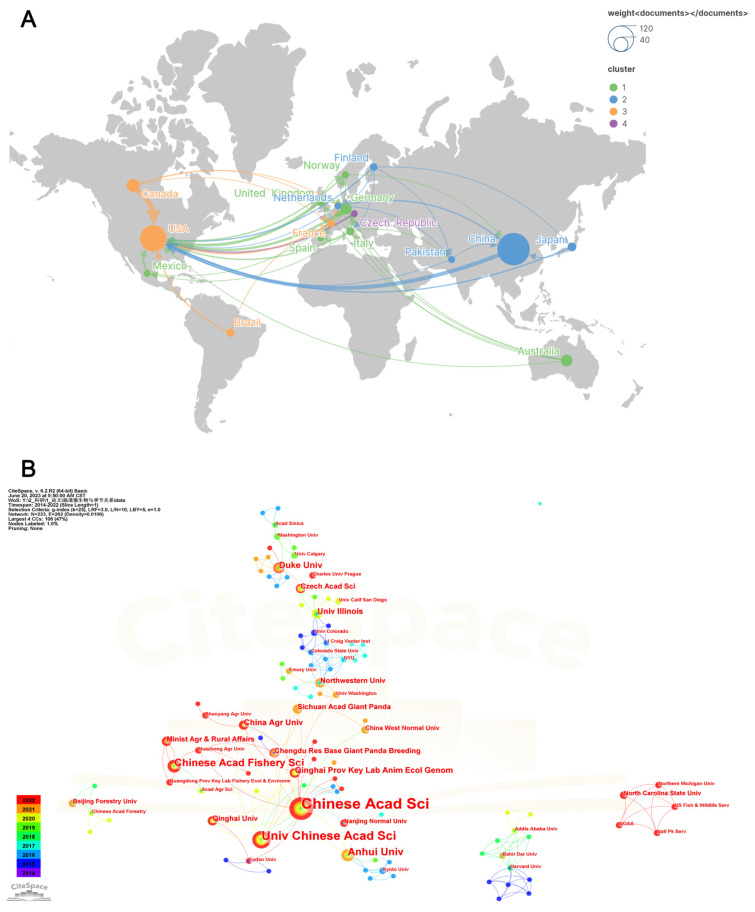
Countries and institutions analysis. (**A**,**B**) Maps of countries and institutions performing research on seasonal variations in gut microbiota, respectively. Node size indicates the number of articles produced. The width of links positively associates with cooperation strength.

Scholars in 63 countries published research on seasonal variations in gut microbiota. The top 20 most productive countries are listed in Table 1. It can be observed that China ranked first, with 112 publications (29.24% of the total), followed by the USA with 74 publications (19.32%), Canada with 18 publications (4.70%), Australia with 14 publications (3.66%), Germany with 12 publications (3.13%), England with 9 publications (2.35%), and Japan with 8 publications (2.09%). As shown in Figure 2A, China and the USA were the central nodes in the international cooperation network, while the USA cooperated the most with other countries, with a total link strength (TLS) of 46, followed by China and Canada with a TLS of 12, Germany with a TLS of 11, England with a TLS of 10, and Italy with a TLS of 9. China and Canada had the closest cooperative relationship with the United States.

A total of 213 institutions participated in research on seasonal variations in gut microbiota and the top 20 most productive institutions are listed in Table 1. China had thirteen, the USA had four, Australia had two and the Czech Republic had one among the top 20 institutions. The Chinese Academy of Sciences ranked first with 30 publications, followed by the University of Chinese Academy of Sciences with 15 publications, Anhui University and Chinese Academy of Fishery Sciences with 9 publications each, and Duke University, The University of Queensland and Royal Brisbane and Women’s Hospital with 6 publications each. The proportion of universities and other institutions among 213 institutions was equivalent, with 111 and 102, respectively. The map of institutions suggests that research cooperation in this field was regional, with domestic cooperation being the focus and international cooperation lacking (Figure 2B).

### 3.3. Journal and Author Analysis

Collaborative networks of journals and authors related to the research on seasonal variations in gut microbiota were operated, and the top 20 journals with the most published articles are shown in Table 2. The most productive journal was *PLoS One* (IF = 3.7), followed by *Applied and Environmental Microbiology* (IF = 4.4), *Proceedings of the National Academy of Sciences of the United States of America* (IF = 11.1), *ISME Journal* (IF = 4.4), *Science* (IF = 56.9), and *Nature* (IF = 64.8).

**Figure 3:**
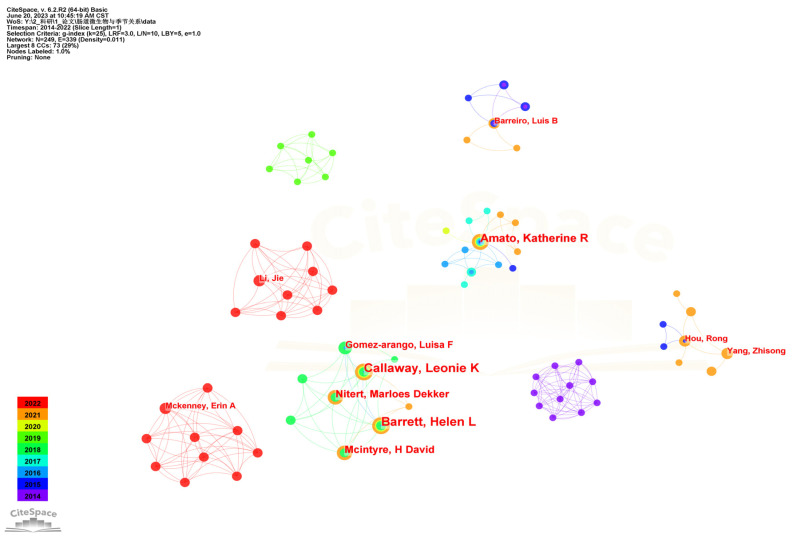
Map of authors performing research on seasonal variations in gut microbiota.

A map of authors related to the seasonal variations in gut microbiota is shown in Figure 3. It can be seen that there were not many connections between authors in this field, forming multiple small groups. Among them, Callaway LK and Barrett HL were more prominent, followed by Amato KR, Nitert MD, and Mcintyre, HD, which reflected their strong academic authority.

### 3.4. Keyword Analysis

**Figure 4:**
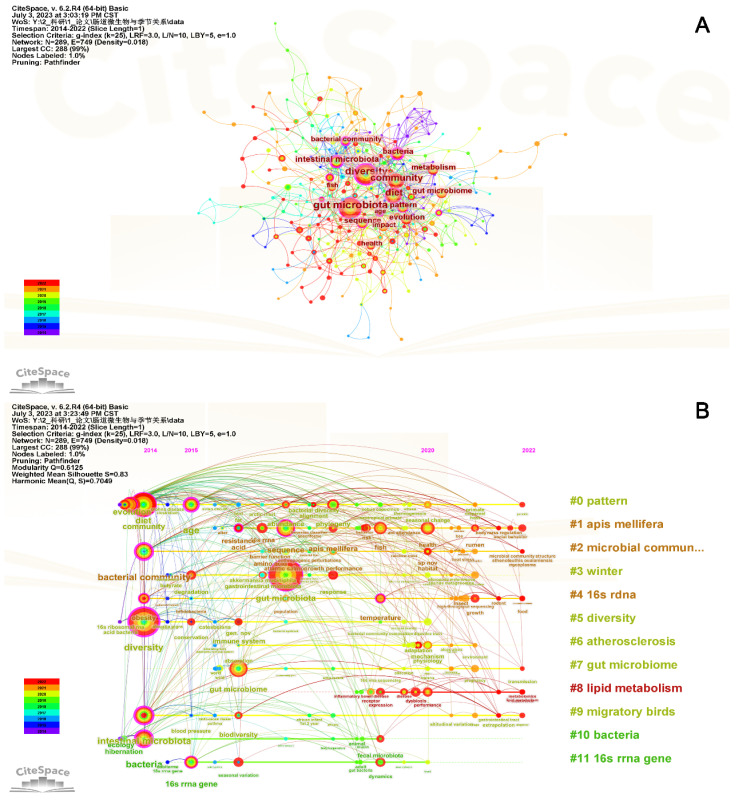
Keyword co-occurrence analysis related to research on seasonal variations in gut microbiota. (**A**) The co-occurring map of keywords. (**B**) The timeline visualization map of keywords.

Keywords summarize the content and reflect the main content and core themes of the literature. In the keywords co-occurrence network (Figure 4A), the larger nodes were for “gut microbiota”, “diversity”, “community”, “diet”, “intestinal microbiota”, “bacteria” and “evolution”. Among them, “gut microbiota” and “diversity” were the two largest nodes, and the connection was also the closest, indicating that research on gut microbiota diversity is a hot topic. Furthermore, studying the diversity of gut microbiota from the perspective of diet is a hot topic needing further attention.

The timeline view was used to demonstrate the changing trends of different clusters over time [25] (Figure 4B). The earliest clusters that appeared were “#0 pattern”, “#5 diversity”, and “#10 bacteria”, while the latest cluster was “#8 lipid metabolism”. The longer durations of “#0 pattern”, “#2 microbial community structure”, “#4 16s rDNA”, and “#9 migratory birds” indicated the importance of these clustering fields, especially “#0 pattern” and “#4 16s rDNA”, which contain more keywords.

**Figure 5:**
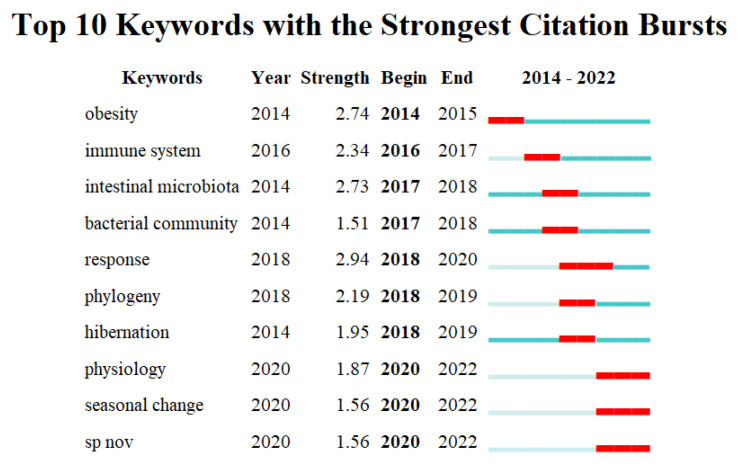
Top 10 keywords with the strongest citation bursts.

Keyword bursts refer to the sharp increase in certain keywords during a certain period, representing changes in cutting-edge topics, research hotspots, and research dynamics in a certain research field [26]. Figure 5 showed 10 keywords with the strongest burst strength in this field. As can be seen, the keywords gradually developed from “obesity”, “immune system”, “intestinal microbiota” and “bacterial community” to the current dimension of “physiology”, “seasonal change” and “sp nov” changes. This indicates that current research is more committed to the relationship between seasonal changes in gut microbiota and host physiology, as well as the research on new strains of gut microbiota.

### 3.5. Analysis of Cited Authors and Co-Cited References

**Figure 6:**
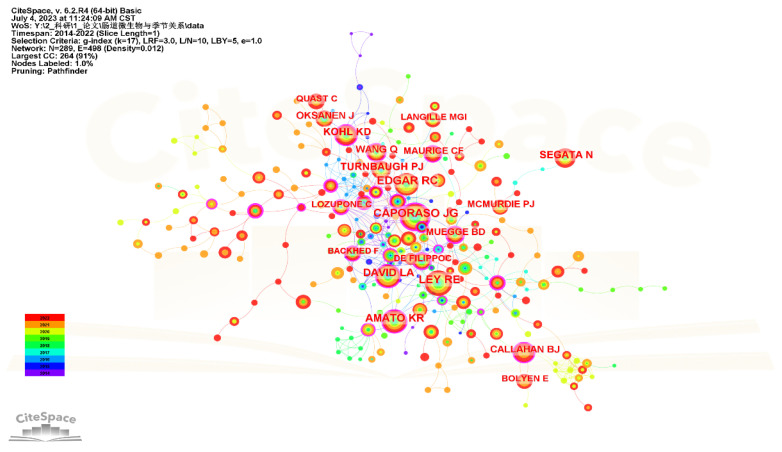
Map of cited authors performing research on seasonal variations in gut microbiota.

Analysis of cited authors aimed to uncover the most active researchers in this field, reflecting their contributions. As shown in Figure 6 and Table 3, the authors with the most citations were Caporaso JG, Ley RE, Edgar RC and Amato KR. Caporaso JG, from Northern Arizona University and Argonne National Laboratory in the USA, described “quantitative insights into microbial ecology” (QIIME) in 2010, which allowed researchers to independently conduct personalized analysis and visualization of gut microbiome amplicon data, gradually becoming the most popular software in this field [27].

**Figure 7:**
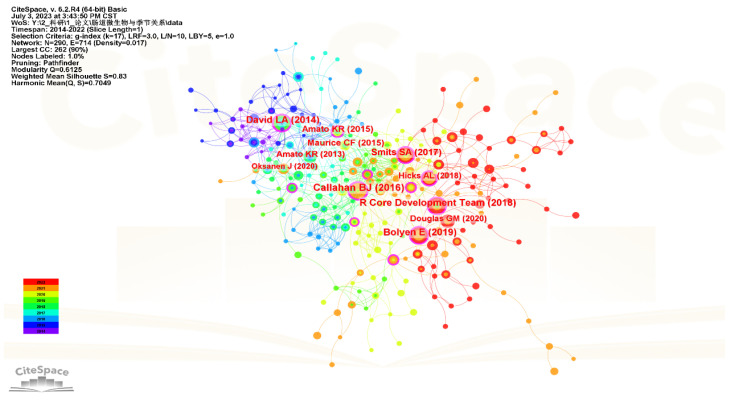
Map of co-cited references related to research on seasonal variations in gut microbiota.

We also revealed the top 10 most highly co-cited references related to research on the seasonal variations in gut microbiota (Table 4), and the visualization map is shown in Figure 7. Among the top 10 co-cited references, there were four references related to research and analysis tools of gut microbiota, which were DADA2, QIIME2, R software, and PICRUSt2 in order of ranking. The remaining six references were about the seasonal variations of gut microbiota in humans, including Hadza hunter–gatherers of Tanzania, wild black howler monkeys (*Alouatta pigra*), wild mice and wild great apes. Among the above six references, the highest cited reference was published in Nature by David LA in 2014, which was about how diet could rapidly and reproducibly alter human gut microbiome, mainly referring to the fact that diet could change the number of gut microbiota and gene expression types within one day [28]. In addition, it is worth noting that 2 of the top 10 highly co-cited references were published by Amato KR in *Isme J* and *Microb Ecol* in 2013 and 2015, respectively, which were both about the seasonal changes in gut microbiota in wild black howler monkey (*Alouatta pigra*) [18,29].

## 4. Discussion

In this study, we retrieved studies on seasonal variations in gut microbiota through the Web of Science Core Collection database, analyzed the number of publications, countries, institutions, journals, authors, and keywords, identified major contributors, and visualized research hotspots and trends in this field over the past few decades. The number of seasonal variations in gut microbiota studies had a general upward trend, and in the past three years (2020–2022) has shown a sharper increase than before, with a higher proportion (65.31%). The trend in citations was similar. Research on the seasonal variations in gut microbiota has become an increasingly popular field [11,12]. The development of technology has promoted the rapid development of this research field, especially high-throughput sequencing technology, which has provided unprecedented insights into the diversity, composition, and function of various microbial ecosystems, including vertebrate intestines, and has completely changed the field of microbial ecology [30].

China contributed the most significant number of publications of any nation, becoming one of the main driving forces of research on seasonal variations in gut microbiota. Obviously, among the top 20 most productive institutions, China also had the highest number of institutions. Chinese Academy of Sciences, as the largest national scientific research institution in China, ranked first in this field, while University of Chinese Academy of Sciences, a comprehensive university born from the Chinese Academy of Sciences to cultivate research talents, followed closely. They mainly focused on species including giant panda (*Ailuropoda melanoleuca*) [31,32], Père David’s deer (*Elaphurus davidianus*) [33], Forest (*Moschus berezovskii*) and Alpine Musk Deer (*Moschus chrysogaster*) [34], Tibetan wild ass (*Equus kiang*) [35], Mongolian gerbils (*Meriones unguiculatus*) [36], Yaks (*Bos grunniens*) [37], Goitered Gazelle (*Gazella subgutturosa*) [38], Brandt’s voles (*Lasiopodomys brandtii*) [39], black-necked cranes (*Grus nigricollis*) [40], Bar-headed geese (*Anser indicus*) [41], greater white-fronted goose (*Anser albifrons*), bean goose (*Anser fabalis*) and swan goose (*Anser cygnoides*) [42,43]. In addition to wintering migratory birds, the species concerned are mainly indigenous species, especially endemic species from China. The USA, represented by Duke University with the highest number of publications in the country, ranked behind China, with research on species such as wild furry-eared dwarf lemurs (*Cheirogaleus crossleyi*) [44].

In terms of journals, researchers focus on *PLoS One*, *Applied and Environmental Microbiology*, *Proceedings of the National Academy of Sciences of the United States of America*, *ISME Journal*, *Science*, and *Nature* for their high-quality studies on seasonal variations in gut microbiota. The author group led by Callaway LK and the author group led by Amato KR were two prominent groups in the author network diagram, respectively studying the gut microbiota of pregnant women and wild Black howler monkeys [18,29,45]. In addition, cited author analysis found that Caporaso JG, Ley RE, Edgar RC and Amato KR were cited the most, indicating that they play a very important role as founders in this research field. As expected, some of them were closely related to the results of co-cited references analysis. Four of the top 10 co-cited references were found to be related to research and analysis tools of gut microbiota, suggesting that these tools provide significant assistance for studying the seasonal variations in gut microbiota. DADA2, a software package that models and corrects Illumina-sequenced amplicon errors, enhanced the study of microbial communities by allowing researchers to accurately reconstruct amplicon-sequenced communities at the highest resolution [46]. Unlike QIIME, which was the most widely used analytical process in the field of microbiome studies, QIIME 2 is a new generation of microbiome comprehensive analysis platform developed based on Python 3 and plug-in architecture to meet the requirements of big data volume and repeated analysis [47]. R software is widely used in various research fields, especially in the study of gut microbiota. PICRUSt2 is a tool developed for prediction of functions from 16S marker sequences, providing interoperability with any operational taxonomic unit (OTU)-picking or denoising algorithm, and enabling phenotype predictions [48]. In addition to the above software tools, the research contents of co-cited references included the seasonal variations of gut microbiota in humans [28], including Hadza hunter–gatherers of Tanzania [49], wild black howler monkeys [18,29], wild mice [11], and wild great apes [50], indicating the high importance and leadership of these studies in the field of seasonal variations in gut microbiota.

Keywords are considered to reflect high-frequency hotspots in specific fields [51]. We conducted a comprehensive analysis of keyword co-occurrence and bursts, and summarized the research hotspots as follows: (1) the diversity of gut microbiota communities in different seasons, such as humans [52] and wild mice [11]; (2) the relationship between diet and gut microbiota in seasonal changes, such as in Mongolians [53] and American Bison [13]; (3) the relationship between gut microbiota and evolutionary adaptation in seasonal changes in giant panda [31,54]. Based on the burst keywords in recent years, we believe that the relationship between physiology and gut microbiota during seasonal changes, the discovery of new strains, and the impact and function of specific strains on seasonal variations in gut microbiota will become academic trends. These will be beneficial for studying how gut microbiota synergistically adapt to seasonal changes, especially the body’s adaptability to cold and heat, as well as further exploring certain dominant microbiota during seasonal changes.

In this study, we combined CiteSpace and VOSviewer to conduct bibliometric analysis for the first time, revealing the hot spots and dynamic changes in the research on seasonal variations in gut microbiota over the past decade. Our study found that the research on seasonal variations in gut microbiota is receiving increasing attention and exhibiting research aggregation characteristics, with multiple academic trends. However, there may be some limitations to our study. Firstly, all articles were only retrieved from the WOSCC database. Even though this is one of the most recognized authoritative databases, the limitation of not including other databases, such as Scopus, China National Knowledge Infrastructure (CNKI) and PubMed, could not be avoided. Secondly, search strategies were not guaranteed to cover all articles. Thirdly, only English articles were selected in this survey, and so non-English articles might have been missed, such as those written in Chinese and Japanese. However, we believe that the results of our analysis are sufficient to reflect the overall state of the research of seasonal variations in gut microbiota over the past decade.

## 5. Conclusions

In summary, the current research provided a beneficial reference to the classic documents and the future trends of the research on seasonal variations in gut microbiota. The number of publications has continued to increase over the last decade and showed a general upward trend. China, USA, Canada, and Australia were the countries that produced the most publications, while the Chinese Academy of Sciences and the University of Chinese Academy of Sciences were the institutions with the most publications. Researchers focused on *PLoS One*, *Applied and Environmental Microbiology*, *Proceedings of the National Academy of Sciences of the United States of America*, *ISME Journal*, *Science*, and *Nature*. Multiple small author groups have been formed, but there is a lack of connection between them. The research hotspots mainly include the diversity of gut microbiota communities in different seasons, the relationship between diet and gut microbiota in seasonal changes, and the relationship between gut microbiota and evolutionary adaptation in seasonal changes. This timely review analyzed the results of the research of seasonal variations in gut microbiota and might advance this field and lay the foundation for future research.

## Figures and Tables

**Table 1:** The top 20 most productive countries and institutions in the research on seasonal variations in gut microbiota.

Rank	Freq	Centrality	Country	Rank	Freq	Centrality	Institution
1	112	0.07	PEOPLES R CHINA	1	30	0.17	Chinese Acad Sci
2	74	0.93	USA	2	15	0.01	Univ Chinese Acad Sci
3	18	0.02	CANADA	3	9	0.04	Anhui Univ
4	14	0.08	AUSTRALIA	4	9	0.02	Chinese Acad Fishery Sci
5	12	0.11	GERMANY	5	6	0.08	Duke Univ
6	9	0.15	ENGLAND	6	6	0	Univ Queensland
7	8	0.08	JAPAN	7	6	0	Royal Brisbane and Womens Hosp
8	7	0.06	ITALY	8	5	0.1	Univ Illinois
9	7	0	BRAZIL	9	5	0	Qinghai Prov Key Lab Anim Ecol Genom
10	6	0.04	FINLAND	10	5	0.01	China Agr Univ
11	6	0.04	NORWAY	11	4	0.13	Northwestern Univ
12	6	0.03	FRANCE	12	4	0.08	Czech Acad Sci
13	5	0.02	NETHERLANDS	13	4	0.01	Chengdu Res Base Giant Panda Breeding
14	5	0.06	PAKISTAN	14	4	0	Sichuan Acad Giant Panda
15	5	0	MEXICO	15	4	0	Qinghai Univ
16	5	0	CZECH REPUBLIC	16	4	0	Chinese Acad Agr Sci
17	5	0	SPAIN	17	4	0	Northeast Agr Univ
18	4	0.03	SWEDEN	18	4	0	Minist Agr and Rural Affairs
19	4	0	ISRAEL	19	3	0	North Carolina State Univ
20	4	0	NEW ZEALAND	20	3	0.01	China West Normal Univ

**Table 2:** Top 20 journals with the most published articles.

Rank	Freq	Centrality	Journal	IF ^1^	Quartile in Category
1	211	0.02	*PLoS One*	3.7	Q2
2	188	0.02	*Appl Environ Microb*	4.4	Q2
3	186	0.03	*P Natl Acad Sci Usa*	11.1	Q1
4	179	0.02	*ISME J*	11	Q1
5	178	0.02	*Science*	56.9	Q1
6	178	0.01	*Nature*	64.8	Q1
7	161	0.02	*Front Microbiol*	5.2	Q2
8	153	0.03	*Sci Rep-UK*	4.6	Q2
9	145	0.03	*Nat Methods*	48.0	Q1
10	130	0.01	*Bioinformatics*	5.8	Q1
11	118	0.02	*Microb Ecol*	3.6	Q1
12	115	0.02	*Microbiome*	15.1	Q1
13	114	0.09	*Mol Ecol*	4.9	Q1
14	114	0.02	*Nucleic Acids Res*	14.9	Q1
15	112	0.02	*Nat Commun*	16.6	Q1
16	96	0.03	*Environ Microbiol*	5.1	Q2
17	94	0.06	*Genome Biol*	12.3	Q1
18	91	0.04	*Nat Rev Microbiol*	88.1	Q1
19	90	0.03	*Fems Microbiol Ecol*	4.2	Q2
20	88	0.14	*Cell*	64.5	Q1

^1^ Data from the 2023 edition of Journal Citation Reports.

**Table 3:** Top 10 cited authors related to research on seasonal variations in gut microbiota.

Rank	Count	Centrality	Cited Authors	Year
1	90	0.23	Caporaso JG	2014
2	86	0.08	Ley RE	2014
3	82	0.06	Edgar RC	2014
4	63	0.11	Amato KR	2015
5	60	0.11	David LA	2014
6	52	0	Segata N	2017
7	50	0.12	Kohl KD	2015
8	49	0.04	Turnbaugh PJ	2014
9	46	0.11	Wang Q	2015
10	46	0.16	Callahan BJ	2018

**Table 4:** Top 10 cited references related to research on seasonal variations in gut microbiota.

Rank	Count	Centrality	Author	Year	Title	Journal
1	29	0.23	Callahan BJ	2016	DADA2: High-resolution sample inference from Illumina amplicon data	*Nat Methods*
2	28	0.15	Bolyen E	2019	Reproducible, interactive, scalable and extensible microbiome data science using QIIME 2	*Nat Biotechnol*
3	27	0.25	David LA	2014	Diet rapidly and reproducibly alters the human gut microbiome	*Nature*
4	26	0.14	R Core Development Team	2018	R: A language and environment for statistical computing	*R Lang Env Stat Comp*
5	23	0.12	Smits SA	2017	Seasonal cycling in the gut microbiome of the Hadza hunter-gatherers of Tanzania	*Science*
6	17	0.35	Amato KR	2015	The gut microbiota appears to compensate for seasonal diet variation in the wild black howler monkey (Alouatta pigra)	*Microb Ecol*
7	17	0.07	Maurice CF	2015	Marked seasonal variation in the wild mouse gut microbiota	*ISME J*
8	16	0.11	Hicks AL	2018	Gut microbiomes of wild great apes fluctuate seasonally in response to diet	*Nat Commun*
9	16	0.01	Douglas GM	2020	PICRUSt2 for prediction of metagenome functions	*Nat Biotechnol*
10	15	0.08	Amato KR	2013	Habitat degradation impacts black howler monkey (Alouatta pigra) gastrointestinal microbiomes	*ISME J*

## Data Availability

Not applicable.
